# Aquaporin-4 expression and modulation in a rat model of post-traumatic syringomyelia

**DOI:** 10.1038/s41598-023-36538-x

**Published:** 2023-06-14

**Authors:** Joel A. Berliner, Magdalena A. Lam, Elmira Najafi, Sarah J. Hemley, Lynne E. Bilston, Marcus A. Stoodley

**Affiliations:** 1 Faculty of Medicine, Health and Human Sciences, Macquarie University, 2 Technology Place, Sydney, NSW 2109 Australia; 2 The ANZAC Research Institute, Concord Repatriation General Hospital, Gate 3, Hospital Road, Sydney, NSW 2139 Australia; 3 Neuroscience Research Australia, Margarete Ainsworth Building, 139 Barker Street, Sydney, NSW 2031 Australia; 4 Faculty of Medicine, School of Clinical Medicine, University of New South Wales, Sydney, NSW 2031 Australia

**Keywords:** Diseases of the nervous system, Spinal cord diseases, Experimental models of disease, Trauma, Neuroscience

## Abstract

Aquaporin-4 (AQP4) has been implicated in post-traumatic syringomyelia (PTS), a disease characterised by the formation of fluid-filled cysts in the spinal cord. This study investigated the expression of AQP4 around a mature cyst (syrinx) and the effect of pharmacomodulation of AQP4 on syrinx size. PTS was induced in male Sprague–Dawley rats by computerized spinal cord impact and subarachnoid kaolin injection. Immunofluorescence of AQP4 was carried out on mature syrinx tissue 12 weeks post-surgery. Increased AQP4 expression corresponded to larger, multiloculated cysts (R^2^ = 0.94), yet no localized changes to AQP4 expression in perivascular regions or the glia limitans were present. In a separate cohort of animals, at 6 weeks post-surgery, an AQP4 agonist (AqF026), antagonist (AqB050), or vehicle was administered daily over 4 days, with MRIs performed before and after the completion of treatment. Histological analysis was performed at 12 weeks post-surgery. Syrinx volume and length were not altered with AQP4 modulation. The correlation between increased AQP4 expression with syrinx area suggests that AQP4 or the glia expressing AQP4 are recruited to regulate water movement. Given this, further investigation should examine AQP4 modulation with dose regimens at earlier time-points after PTS induction, as these may alter the course of syrinx development.

## Introduction

Syringomyelia occurs in nearly one third of patients with spinal trauma^1–4^, consisting of a fluid-filled cyst (syrinx) at or near the site of injury. PTS cavities are lined by astrocytic processes without ependymal involvement and develop anywhere from a few weeks to decades post-injury^5–9^. As the syrinx expands it compresses the surrounding spinal cord, causing pain, weakness or paralysis, sensory loss, or respiratory arrest^3,10,11^. The pathogenesis of syrinx formation remains unknown^12^. Consequently, surgical treatments do not necessarily correct the underlying pathophysiology. Suboptimal long-term outcomes result, with syrinxes recurring in up to 50% of post-surgical cases^6^. To improve treatment outcomes and develop non-surgical therapies we need greater knowledge of the hydrodynamics of syrinx etiology.

The water channel protein, AQP4, primarily expressed on astrocytes, is involved in the regulation of water movement in the central nervous system^13,14^. Changes to AQP4 expression have been implicated in fluid accumulation disorders. AQP4 upregulation and/or its relocalization may either worsen, or aid in the elimination of, edema resulting from conditions such as cerebral contusion, hydrocephalus, bacterial meningitis, subarachnoid hemorrhage, and spinal cord injury^15–21^. In the early stages of spinal cord injury in rats, upregulation of AQP4 promotes the clearance of vasogenic edema, yet in later stages AQP4 exacerbates cytotoxic edema^20^. Inhibition or relocalization of AQP4 has been shown to ameliorate motor deficits and reduce cytotoxic edema post-injury^20–22^. Deletion of AQP4 in mice has also been shown to reduce spinal cord edema after short compressive injury^23^. In an excitotoxic injury model of syringomyelia, AQP4 was upregulated around the enlarging syrinx 3 and 6 weeks post-surgery, which suggests AQP4 could play a critical role in PTS. However, whether it is contributing to the expansion of, or aiding to resolve the fluid accumulation within the syrinx is unknown^24^.

Modulation of AQP4 activity holds potential as a therapy for fluid imbalance disorders of the central nervous system. Pre-treatment with an AQP4 antagonist, TGN-020, in a mouse brain model of focal ischemia was reported to significantly diminish infarct volume and edema^25^. In a rat model of non-reperfusion ischemia, a single dose of the AQP4 antagonist after ischemia induction significantly diminished edema and glial scarring^26^. Similarly, concurrent pre-treatment with TGN-020 and a Na^+^-K^+^-Cl^−^ cotransporter 1 (NKCC1) antagonist in a rat model of spinal cord contusion reduced spinal cord edema, lesion size, and the expression of both AQP4 and NKCC1^27^. The following study examined whether the expression of AQP4 increased around perivascular spaces and syrinx borders in PTS, and whether agonism or antagonism of AQP4 in PTS could ameliorate syrinx progression.

## Results

### AQP4 immunofluorescence at syrinx border

**Figure 1:**
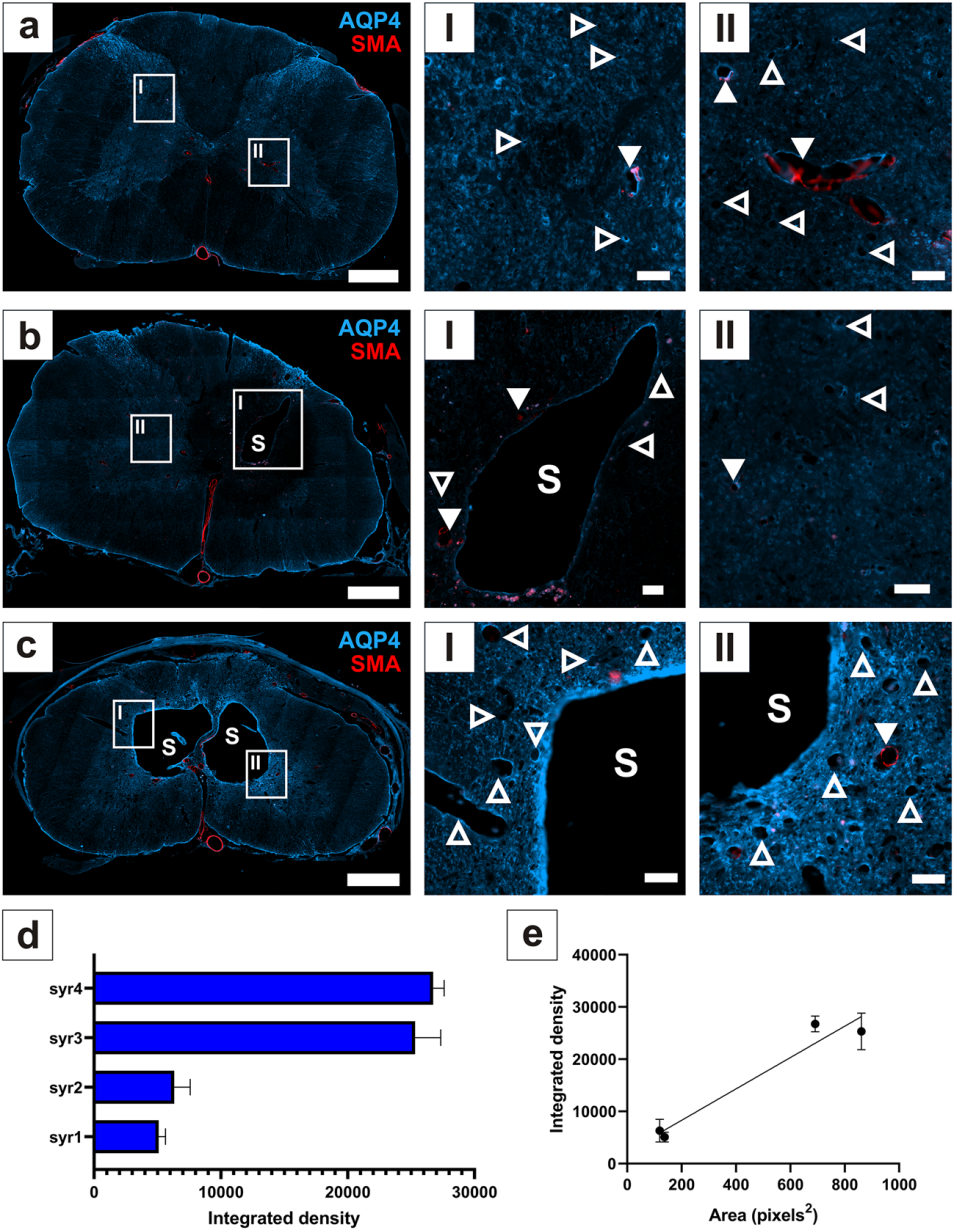
AQP4 expression at the syrinx border varies with syrinx size. Immunofluorescence micrographs in (**a**) show AQP4 and smooth muscle actin (SMA) expression in a control spinal cord, with grey matter region insets (I, II). Micrographs in (**b**, **c**) indicate that syrinx size can vary at the same time-point of 12 weeks after PTS induction, where a single cavity (**b**) or multiloculated cavities (**c**) are present. Insets (I, II) of (**b**, **c**) show that expression of AQP4 at the syrinx border differs between syrinx cavities. (**d**) There was considerable variation in expression of AQP4 at the border of these syrinx cavities (n = 4), reported as integrated density (mean ± SD). (**e**) Increased AQP4 expression is strongly associated with increased syrinx area, R^2^ = 0.94, *p* = 0.029 (Pearson Correlation test, mean ± SD). Arrowheads: arterioles, open arrowheads: capillaries and venules. Scale bars are 500 µm (**a**–**c**) and 50 µm (I, II).

Prominent AQP4 expression was found in the grey matter of control spinal cords (Fig. 1a). AQP4 was expressed at low levels around small syrinx cavities (Fig. 1b), but was higher along the border of large, multiloculated syrinx cavities (Fig. 1c). There was considerable variation in AQP4 expression around syrinx cavities (Fig. 1d) Larger syrinx size was strongly associated with higher AQP4 expression around the syrinx (linear regression, slope, R^2^ = 0.94, *p* = 0.029) (Fig. 1e).

### AQP4 expression by immunoelectron microscopy

#### Arterioles

**Figure 2:**
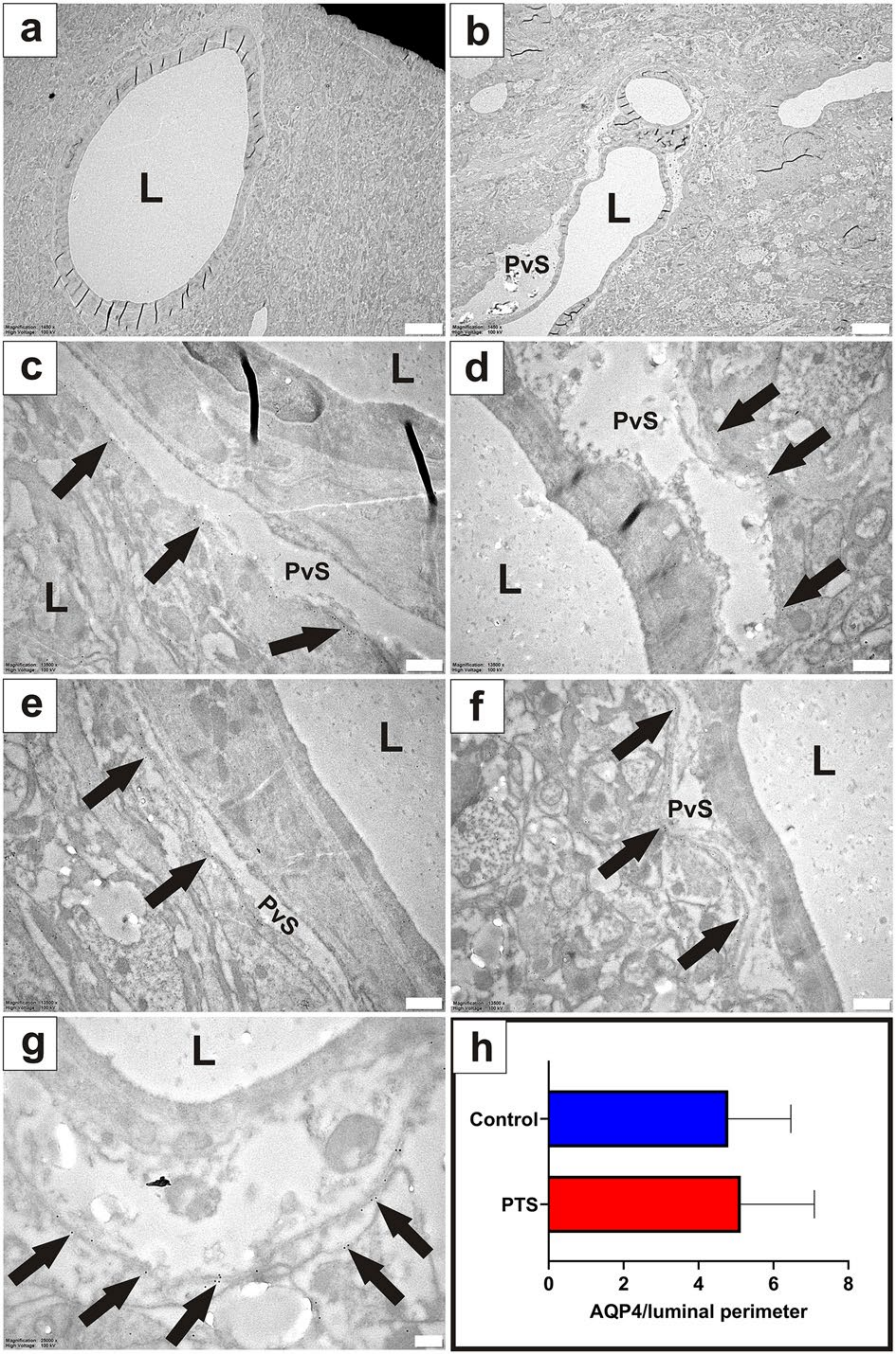
There is no difference in AQP4 expression around arterioles between control and PTS, despite structural changes. Arterioles of the grey matter were phenotypically normal in control animals (**a**) and typically exhibited an enlarged perivascular space (PvS) in PTS animals (**b**). Spherical gold nanoparticles (10 nm) bound to antibodies for AQP4 showed consistent expression along the concentric border of arterioles, irrespective of a normal (**c**, **e**) or an enlarged (**d**, **f**) perivascular space. Higher magnification view (**g**) details the location of the immuno-labelled AQP4 with 10 nm spherical gold nanoparticles (arrows) on the foot processes of astrocytes. Expression of AQP4 along the entire border of arteriole profiles was quantified using ImageJ and expressed as a ratio to the luminal perimeter (**h**). Results in (**h**) are mean ± SD (n = 4 arterioles/replicate per animal; unpaired t-test, *p* = 0.803). L: lumen. Scale bars are 5 µm (**a**, **b**), 500 nm (**c**–**f**), and 200 nm (**g**).

At the level of the syrinx, arterioles were surrounded by an enlarged perivascular space (Fig. 2b) compared to control tissue (Fig. 2a). Immunogold labelling for AQP4 showed preferential binding to the apical membrane of astrocytic foot processes lining the perivascular space in both control and PTS tissue (Fig. 2c–g). No statistical differences were found for the expression of AQP4 around arterioles between control and PTS cohorts (unpaired t-test, *p* = 0.803; Fig. 2h).

#### Venules

**Figure 3:**
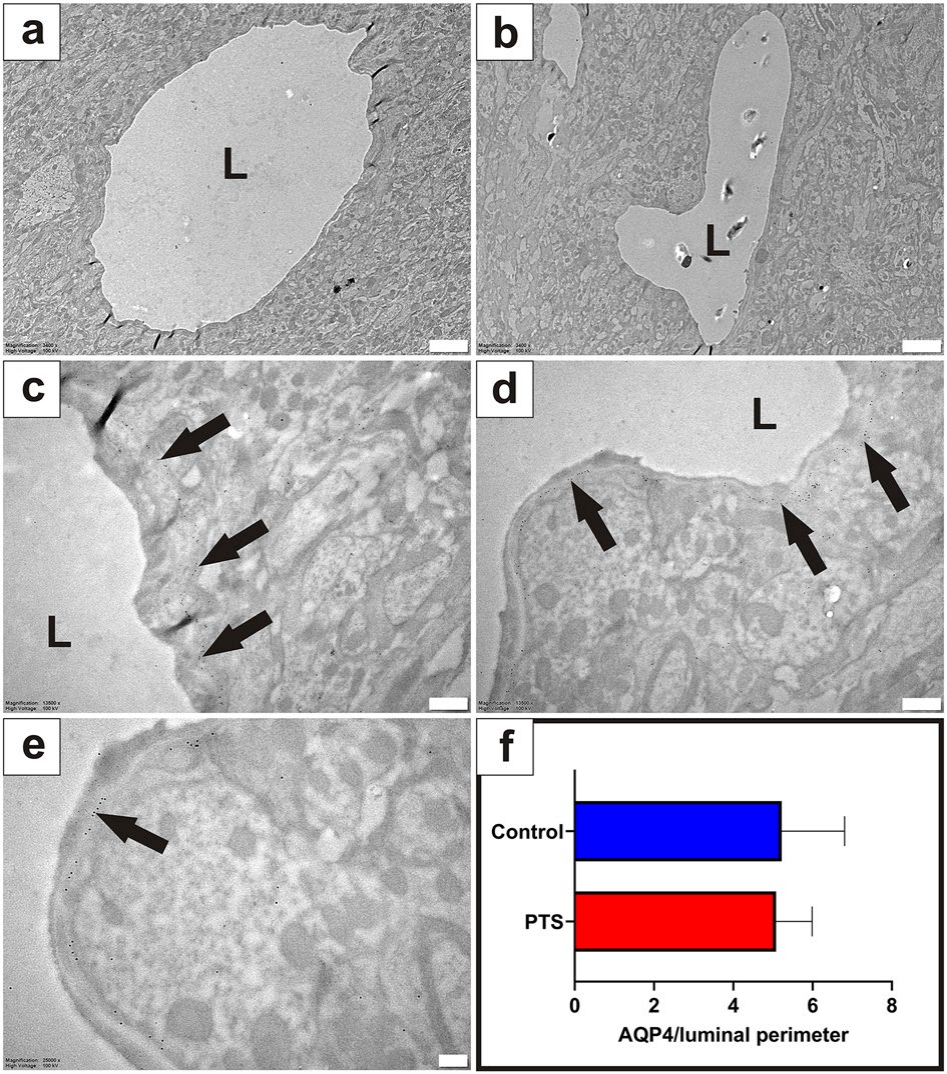
There is no difference in AQP4 expression around venules in control and PTS tissue. Venules of the grey matter were phenotypically normal in control animals (**a**) and PTS animals (**b**). Spherical gold nanoparticles (10 nm) bound to antibodies for AQP4 showed consistent expression along the concentric border of venules in control (**c**) and PTS (**d**) tissue. Higher magnification view (**e**) details the location of the immuno-labelled AQP4 with 10 nm spherical gold nanoparticles (arrow) on the foot processes of astrocytes. Expression of AQP4 along the entire border of venule profiles was quantified using ImageJ and expressed as a ratio to the luminal perimeter (**f**). Results in (**f**) are mean ± SD (n = 7 venules/replicate per animal; unpaired t-test, *p* = 0.882). L: lumen. Scale bars are 2 µm (**a**, **b**), 500 nm (**c**, **d**), and 200 nm (**e**).

Venules did not appear to have enlarged perivascular spaces in the PTS cohort compared to the controls (Fig. 3a,b). Immunostaining for AQP4 was similar around venules and arterioles, with dense AQP4 staining at the apical membrane of astrocytic foot processes lining the thin perivascular space/basal lamina of venules in control and PTS tissue (Fig. 3c–e). Expression of AQP4 was not statistically different around venules between control and PTS cohorts (unpaired t-test, *p* = 0.882; Fig. 3f).

#### Capillaries

**Figure 4:**
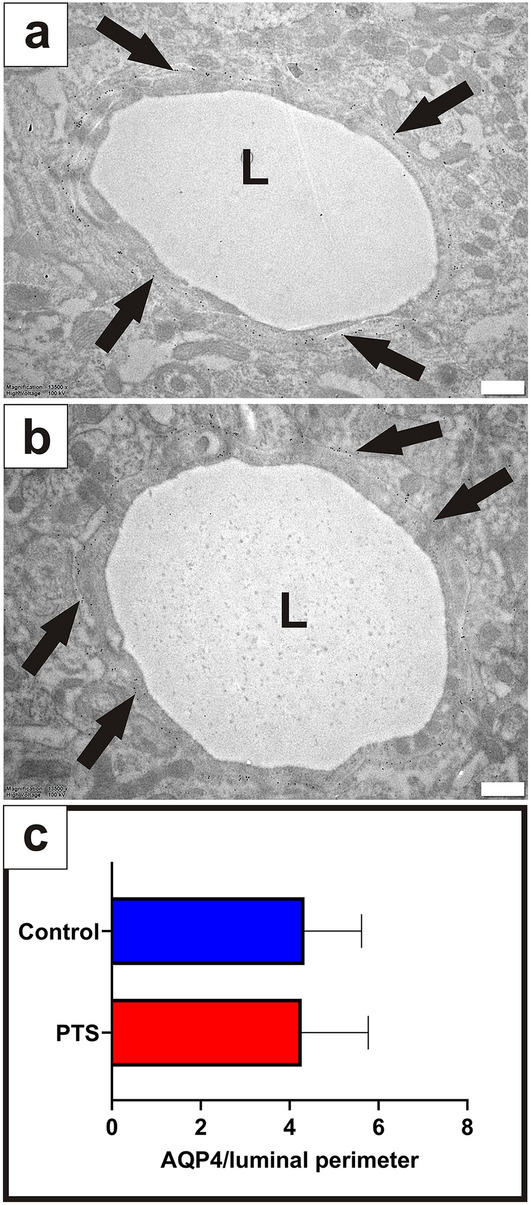
There was no difference in AQP4 expression around capillaries in control and PTS tissue. Capillaries were phenotypically normal across all animals, and spherical gold nanoparticles (10 nm) bound to antibodies for AQP4 (arrows) showed consistent expression along the concentric border of capillaries in control (**a**) and PTS (**b**) tissue. Expression of AQP4 along the entire border of capillary profiles was quantified using ImageJ and expressed as a ratio to the luminal perimeter (**c**). Results in (**c**) are mean ± SD (n = 28 capillaries/replicate per animal; unpaired t-test, *p* = 0.955). L: lumen. Scale bars are 500 nm (**a**, **b**).

Capillaries were structurally similar in control and PTS tissue (Fig. 4a,b). AQP4 immunogold labelling demonstrated a similar pattern to arterioles and venules, localized to the apical membrane of astrocytic foot processes lining the basal lamina. AQP4 expression was statistically similar around capillaries in control and PTS tissue (unpaired t-test, *p* = 0.955, Fig. 4c).

#### Glia limitans

**Figure 5:**
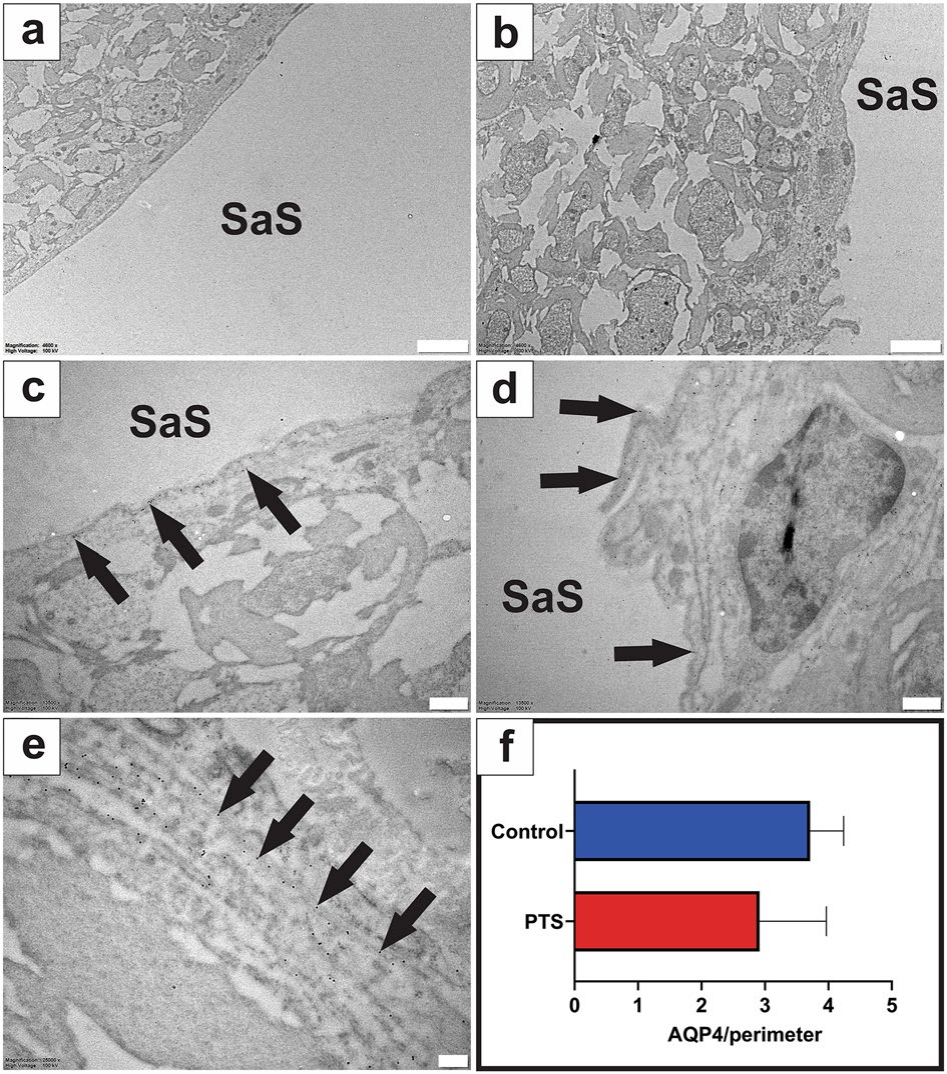
Expression of AQP4 at the glia limitans was unchanged in PTS tissue when compared to controls. The glia limitans appeared normal in control (**a**) and PTS animals (**b**), and spherical gold nanoparticles (10 nm) bound to antibodies for AQP4 showed consistent expression along the astrocytic border interfacing with the subarachnoid space (SaS) in control (**c**) and PTS (**d**) tissue. Higher magnification view (**e**) details the location of the immuno-labelled AQP4 with 10 nm spherical gold nanoparticles (arrows) on the processes of astrocytes. Results in (**f**) are mean ± SD (n = 17 glia limitans/replicate per animal; unpaired t-test, *p* = 0.225). Scale bars are 2 µm (**a**, **b**), 500 nm (**c**, **d**), and 200 nm (**e**).

The morphology of the astrocytes at the glia limitans was similar in both PTS and control animals (Fig. 5a,b). AQP4 was expressed on the astrocytic processes facing the subarachnoid space. This was variable along the length of the glia limitans in both control and PTS spinal cords (Fig. 5c–e), but overall density of AQP4 expression was comparable between control and PTS animals (unpaired t-test, *p* = 0.225; Fig. 5f).

### Effect of AQP4 activity modulation on syrinx size

#### MRI analysis of syrinx size

**Figure 6:**
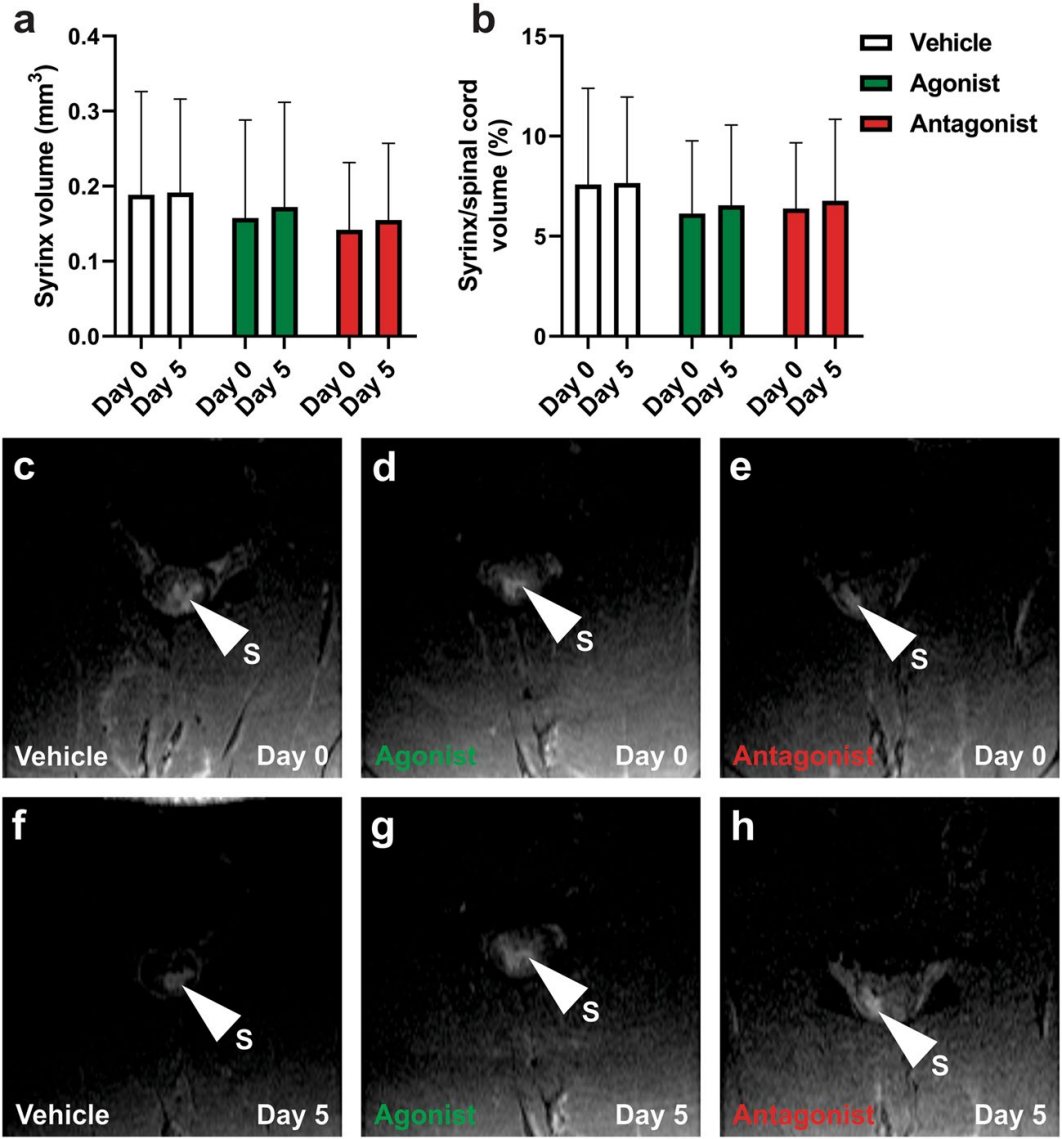
Syrinx volume quantification on MRI before and after modulation of AQP4 activity. (**a**) Absolute syrinx volume and (**b**) syrinx volume as a percentage of the respective whole spinal cord volume were taken before (day 0) and after (day 5) modulation with AqF026 (agonist; n = 9), AqB050, (antagonist; n = 10) and DMSO in saline (vehicle; n = 10). (**c**–**h**) Representative MRIs in the axial plane for vehicle (**c**, **f**), agonist (**d**, **g**) and antagonist (**e, h**) at day 0 (**c**–**e**) and day 5 (**f**–**h**). The white arrowhead indicates the syrinx (S), which appears as a hyperintense region on T2-weighted MRI. Results in (**a**) are mean ± SD (two-way ANOVA, *p* = 0.738) and (**b**) are mean percentage ± SD (two-way ANOVA, *p* = 0.790).

**Figure 7:**
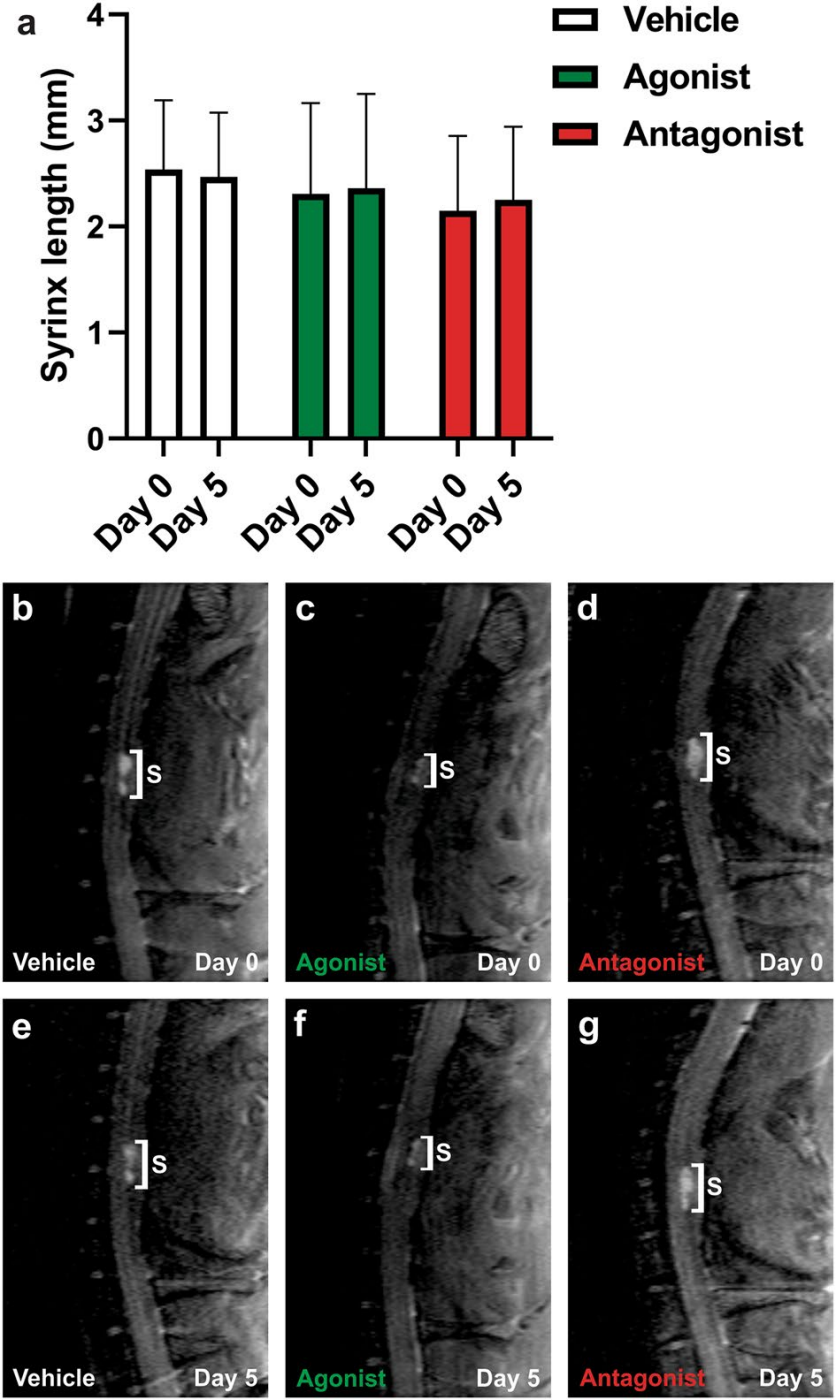
Syrinx length measurements on MRI before and after modulation of AQP4 activity. (**a**) Syrinx length is shown before (day 0) and after (day 5) modulation with AqF026 (agonist; n = 9), AqB050 (antagonist; n = 10) and DMSO in saline (vehicle; n = 10). (**b**–**g**) Representative MRIs in the sagittal plane for vehicle (**b, e**), agonist (**c, f**) and antagonist (**d, g**) at day 0 (**b**–**d**) and day 5 (**e**–**g**). The white bar indicates the syrinx (**S**), which appears as a hyperintense region on the T2-weighted MRI of the spinal cord. Results in (**a**) are shown as mean ± SD (two-way ANOVA, *p* = 0.882).

Prior to AQP4 modulation (day 0), hyperintense regions (syrinx) were visible in both axial (Fig. 6c–e) and sagittal (Fig. 7b–d) images. Corresponding axial (Fig. 6f–h) and sagittal (Fig. 7e–g) images at day 5, after AQP4 modulation, also had distinct hyperintense regions. Syrinx cavities were most frequently seen spanning the grey matter and into the dorsal white matter. The CSF-filled subarachnoid space was visible as a hyperintense region around the spinal cord (Fig. 6f).

Syrinx volume did not change significantly pre- and post-modulation in any of the three treatment groups (vehicle, AQP4 agonist, AQP4 antagonist; Fig. 6a, two-way ANOVA, *p* = 0.738; Fig. 6b, two-way ANOVA, *p* = 0.790). Syrinx length did not change significantly following AQP4 modulation (Fig. 7a, two-way ANOVA, *p* = 0.882).

#### Histological analysis of syrinx size

**Figure 8:**
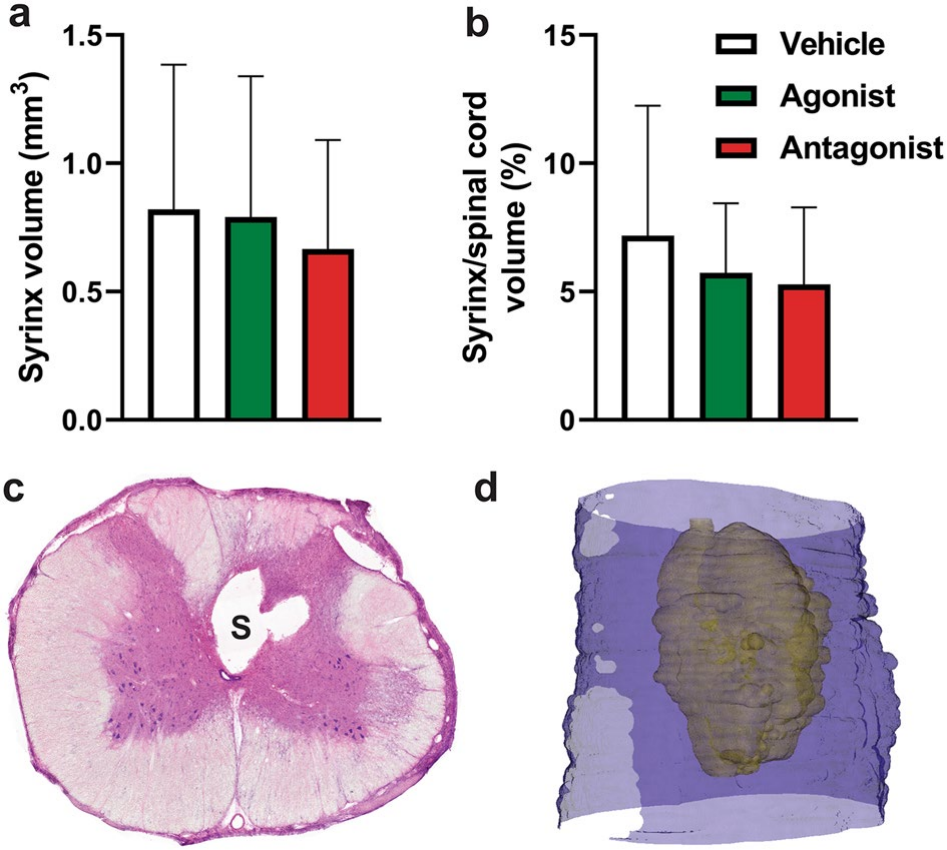
Syrinx volume quantification from histological sections 6 weeks after the commencement of AQP4 modulation. (**a**) Absolute syrinx volume and (**b**) syrinx volume as a percentage of the respective whole spinal cord volume were measured following AQP4 modulation with AqF026 (agonist; n = 9), AqB050 (antagonist; n = 10) and DMSO in saline (vehicle; n = 10). (**c**) Representative hematoxylin and eosin (H&E) stained cross-section of the spinal cord with a clearly demarcated syrinx (S) and compressed central canal. (**d**) Serial H&E sections of the syrinx were volume rendered for the cavity and the whole cord using a combination of ImageJ and Python software to provide a 3D representation of the syrinx cavity in situ. Total syrinx images were captured in 3D Slicer using Linux. Results in (**a**) are mean ± SD (one-way ANOVA, *p* = 0.780) and (**b**) are mean percentage ± SD (one-way ANOVA, *p* = 0.520).

Morphological changes to the spinal cord parenchyma were in line with previous reports^28^. This included the presence of edema and hemorrhage, as well as astrocyte migration to the syrinx border. A combination of high-density astroglial scarring around the syrinx cavities with diffuse, low-density parenchyma adjacent to the cavities was a typical observation. Syrinx cavities presented as either one large cavity, or as multiloculated cavities separated by glial septa. The central canal was generally not involved in the syrinx and appeared compressed (Fig. 8c) or heavily dilated near the syrinx. When invaginated and included in the syrinx, localized ependyma intermixed with astroglia to form part of the syrinx border.

**Figure 9:**
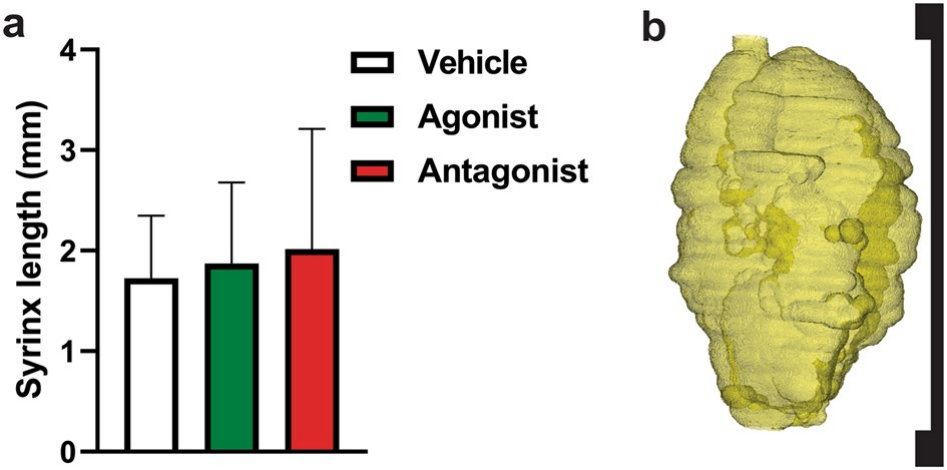
Syrinx length quantification from histological sections 6 weeks after the commencement of AQP4 modulation (**a**) Syrinx length in animals following AQP4 modulation with AqF026 (agonist; n = 9), AqB050 (antagonist; n = 10) and DMSO in saline (vehicle; n = 10). (**b**) Volume rendered syrinx showing bar bracket indicating length of cavity. Results in (**a**) are shown as mean percentage ± SD (one-way ANOVA, *p* = 0.779).

Syrinx volume was not significantly altered by AQP modulation, either for absolute syrinx volume (Fig. 8a, one-way ANOVA, *p* = 0.780) or volume as a percentage of the whole cord (Fig. 8b, one-way ANOVA, *p* = 0.520). A 3D volume reconstruction of a typical syrinx cavity is shown in Fig. 8d. Histological analysis showed no significant differences in syrinx length between experimental groups (Fig. 9a, one-way ANOVA, *p* = 0.779). Syrinx length is represented by the syrinx reconstruction and bar bracket in Fig. 9b.

## Discussion

This study examined AQP4 expression in an animal model of PTS and the effect of modulating AQP4 activity on syrinx size. Immunofluorescence analysis demonstrated that AQP4 expression at the syrinx border varied and was strongly linked to cavity size. However, increasing or inhibiting AQP4 activity did not change syrinx size. Immunogold labelling did not demonstrate any differences in AQP4 expression on astrocytic foot processes surrounding perivascular spaces or at the glia limitans in animals with PTS when compared with control tissue.

Expression of AQP4 at the syrinx border was strongly associated with syrinx size. This increase surrounding the syrinx likely reflects glial scar formation and may be interpreted in one of two ways. As syrinx size increases, more reactive astrocytes migrate to the glial scar. The increase in AQP4 levels reflects the increased number of reactive astrocytes. Alternatively, as more reactive astrocytes migrate to the glial scar, syrinx size increases. AQP4 has been demonstrated to promote glial scarring^29^. In vivo assessment of AQP4-null astrocytes in mice has shown that astrocyte migration and glial scar formation are significantly reduced when AQP4 is absent^30^. Several studies in rat spinal cord injury by Nesic et al*.*^31,32^ have suggested that the persistent upregulation of AQP4 demonstrated in chronic spinal cord injury could reflect astrocyte migration to the site of trauma. Interestingly when measured against GFAP expression, the trend of AQP4 upregulation around syrinx cavities was less evident over time, showing significance at 6 weeks but not 12 weeks post-surgery. Increased expression around the syrinx cavities was likely due to the recruitment of more astroglia to the glial scar, rather than astrocytic overexpression of AQP4^24^. The association between syrinx size and AQP4 expression observed here may indicate an attempt by astroglia to draw water from the growing syrinx or a pathway for water ingress to the syrinx. Investigation of glial scar formation following AQP4 modulation may be useful in determining the role of glial scarring in the development or enlargement of PTS. Irrespective of whether AQP4 here holds an adverse or protective function, modulation of its activity was expected to yield changes to syrinx size.

In the present study, syrinx size was unaltered by AQP4 modulation. This contrasts with other studies, where direct and indirect modulation of AQP4 have been shown to alter the course of other fluid accumulation pathologies. In a rat cortical contusion injury model, indirect suppression of AQP4 by the vasopressin 1a (V1a) receptor antagonist SR49059 resulted in decreased brain water content in the contused hemisphere^33^. Similarly, activation of Protein Kinase C by phorbol ester has been shown to indirectly close AQP4 channels and significantly reduce brain water content after traumatic brain injury in rats^34^. The direct antagonism of AQP4 has also proven successful in alleviating hallmark pathologies in rat models of nonreperfusion ischemic stroke^26,35^ and spinal cord compression^36^. A single, intraperitoneal injection of the potent antagonist for AQP4, TGN020, significantly reduced cerebral edema, the extravasation of serum albumin, apoptotic events, and glial scarring at 3 and 7 days after occlusion of the middle cerebral artery^26,35^. Comparable results were observed in a spinal cord compression model, where a single intraperitoneal dose of TGN020 promoted functional recovery at 3, 7, 14, 21, and 28 days postinjury, and significantly reduced edema, gliosis and AQP4 expression at 3 days^36^. These studies, although promising, commenced AQP4 modulation within 15 min of injury or stroke—time-points which are not clinically feasible.

The relative timing of pharmacomodulation of AQP4 is an important consideration in this study. It is clear from the literature that the timing of administration of commercially available antagonists for AQP4 has a profound effect on edema resolution. In the present study, the aim was to investigate whether AQP4 is a potential therapeutic target for PTS. To be clinically relevant, AQP4 modulation would need to be administered post-injury, rather than as a preventative measure. The use of the AQP4 modulators in this study was based on previous data from a traumatic brain injury model in mice^37^ where a single tail vein injection of AqF026 (0.2 mg/kg) and AqB013 (0.8 mg/kg, pharmacologically synonymous to AqB050) successfully reduced cerebral edema. The timing of AQP4 modulator administration at 6 weeks after PTS induction surgery was based on acquired data from the excitotoxic induction of PTS^24^, which showed that at 6 weeks there was an increase in AQP4 expression. The application of pharmacological modulators for AQP4 at an earlier time-point may elicit significant changes to AQP4 expression at stages critical to syrinx development. We cannot rule out the possibility that the route of administration in the present study resulted in an inadequate concentration for modulation of AQP4. Other concentrations and routes of administration (intravenous and/or intracisternal) need to be tested to definitively determine the utility of AQP4 modulation in PTS. Application of a commercially available inhibitor for AQP4^22,25,36,38^ in this model of PTS would also be an appropriate step to validate these results. Astrocytes possess a number of ion channels apart from AQP4 (such as the co-transporter NKCC1) that can transfer water across the plasma membrane in response to osmotic gradients^39^. The concurrent administration of antagonists for AQP4 and NKCC1, which has previously been applied as a pre-treatment to spinal cord injury in rats^27^, could be useful for reducing syrinx development and progression. Another useful target for reducing fluid accumulation in PTS may be the subcellular calmodulin-mediated AQP4 localization pathway. In a spinal cord injury model, Kitchen et al*.*^22^ were able to ablate edema formation and reduce AQP4 localization post-injury by direct intralesional inhibition of calmodulin. Applied to the present model of PTS, inhibiting this pathway of AQP4 localization may be able to attenuate syrinx development.

The lack of significant change in syrinx volume reported in this study may be due to the complexity of this pathology, including the contribution of two counteractive elements—cytotoxic edema and vasogenic edema—and the respective ways AQP4 results in or resolves each process. AQP4 antagonism could ameliorate the effects of cytotoxic edema by reducing fluid accumulation intracellularly, yet by limiting the activity of AQP4 channels, excess extracellular fluid accumulation cannot be removed across the glia limitans, thereby engorging the extracellular space; conversely, AQP4 agonism could ameliorate the extracellular accumulation of fluid (vasogenic edema) resulting from a damaged BSCB, which has been reported at 6 weeks after PTS induction in rats^40^, yet by increasing AQP4 activity, may exacerbate cell swelling (cytotoxic edema). It is possible that if both edema processes occur synchronously in the pathogenesis of syrinx formation and enlargement, then modulation of AQP4 may not result in an overall change in fluid accumulation.

In the present study, there were no clear changes in AQP4 expression at perivascular locations adjacent to the syrinx cavity or at the glia limitans. It is possible that fluid is entering the syrinx via perivascular pathways independent of AQP4. In the normal spinal cord, AQP4 may facilitate the movement of water from perivascular spaces into the parenchyma, as described in the brain^29,41^. However, when the spinal subarachnoid space is obstructed (under conditions of injury or inflammation), the amount of fluid influx into the spinal cord increases^42^. Pathological enlargement of perivascular spaces and cavitation of the basal lamina around capillaries has been revealed in a rat model of PTS, where it is suggested that disruptions to subarachnoid flow may result in and worsen this phenotype^43^. The enlargement of fluid spaces around vessels may overwhelm the AQP4-dependent flow through astrocytes^44^, and fluid may move between intercellular gap junctions to expand the extracellular space.

## Conclusion

AQP4 expression is unaltered at CSF-facing glial boundaries in mature syrinxes yet is enriched at the glia surrounding large complex cavities. Although no statistically significant differences in syrinx volume were observed when AQP4 activity was modulated, AQP4 may still play a role in syrinx development and enlargement at other time-points. Further investigation is warranted.

## Methods

**Figure 10:**
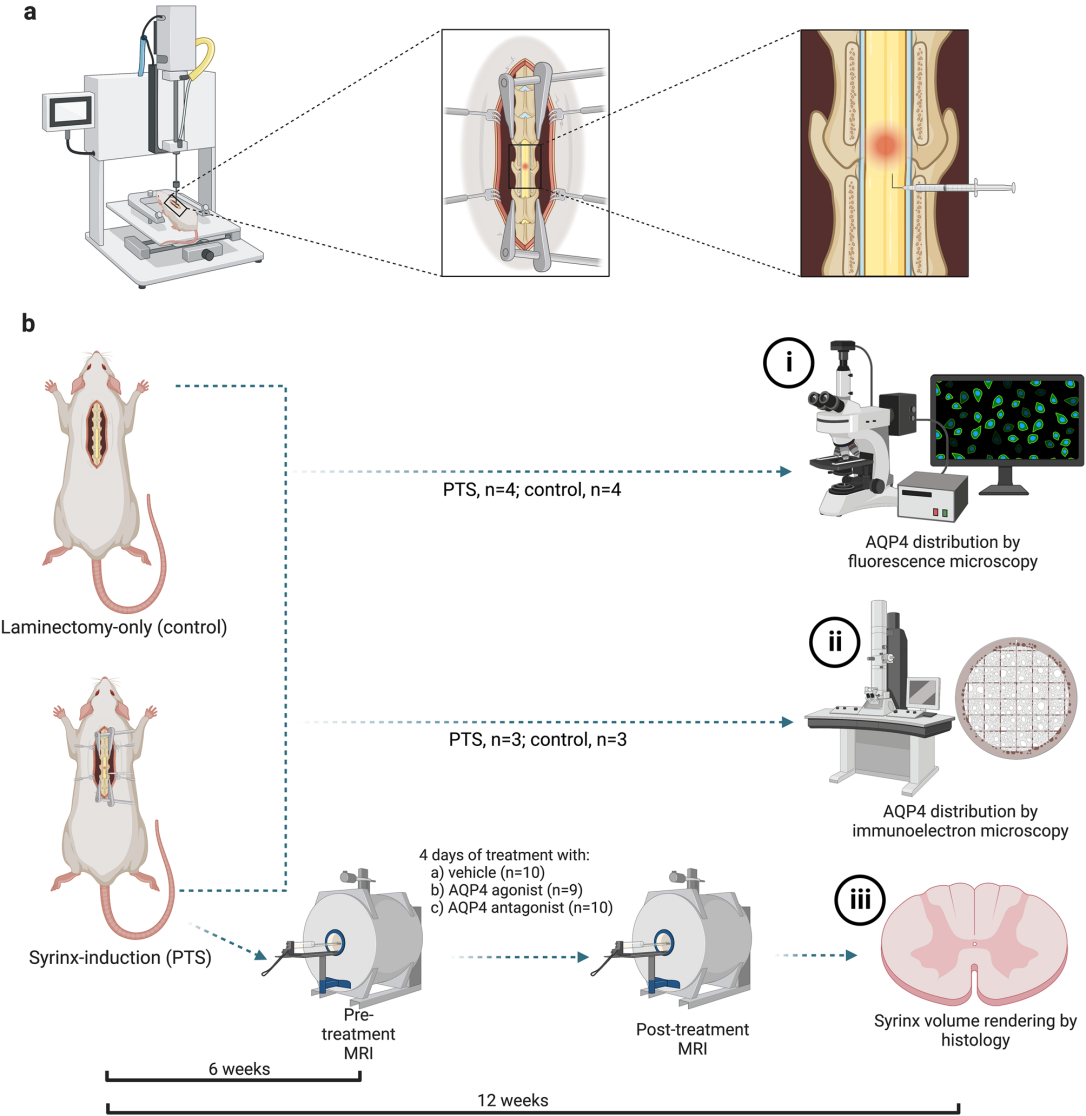
Experimental timeline for AQP4 studies in PTS. (**a**) In 5–7-week-old Sprague–Dawley rats, a syrinx was induced between C6 and C8 spinal cord levels by a computer-controlled impact to the spinal cord, followed by a subarachnoid injection of kaolin dissolved in saline. (**b**) In two separate cohorts 12 weeks post-surgery, AQP4 expression was assessed by (i) immunofluorescence and (ii) immunoelectron microscopy. (iii) In the final cohort 6 weeks after surgery, MRI was performed to detect the syrinx cavity, followed by daily injections of an AQP4 modulator or vehicle over 4 consecutive days. A second MRI was performed 5 days after the first MRI. Twelve weeks after syrinx-induction surgery, syrinx cavities were histologically assessed. Created with BioRender.com.

This study was carried out in accordance with the Australian Code for the Care and Use of Animals for Scientific Purposes and reported in accordance with ARRIVE guidelines^45^. Experimental procedures were approved by Macquarie University Animal Ethics Committee (Protocol No. 2016/032) and University of New South Wales Animal Care and Ethics Committee (Protocol No. 17/45B). At 5–7 weeks of age, 44 male Sprague–Dawley rats weighing 359 ± 42 g (mean ± SD) underwent PTS or control surgery following an established model (Fig. 10a)^46^. The three arms of the study investigated: AQP4 expression by (i) immunofluorescence and (ii) immunogold labelling, and (iii) the effect of AQP4 modulation on syrinx size (Fig. 10b). Since the aim of this study was to determine the therapeutic potential of AQP4, the agonist and antagoniost were administered in a model of post-traumatic syringomyelia at 6 weeks after induction.

Post-operatively, animals were monitored for excessive weight loss, limb weakness, urinary retention, or excessive self-grooming. One animal required a 5-day course of cephazolin (50 mg/kg) after a wound infection was discovered when sutures were removed. Dry feed and water were supplied ad libitum. One animal failed to develop syringomyelia (based on MRI) and was excluded from further analysis.

### PTS model

Anesthesia was induced with 5% isoflurane in oxygen (1 L/min) and maintained with 2–3% isoflurane in oxygen (0.2 L/min) through a nose cone. With the animal in the prone position, the skin was shaved and prepared with chlorhexidine and povidone iodine. An incision was made over the cervicothoracic junction and a C6–C7 laminectomy performed. At this point, laminectomy-only (control) animals were procedurally complete and the wound was closed. For PTS animals, the C5 and T1 laminae were exposed and the spinal column secured using twin forceps. A force of 75 kdyn was delivered to the spinal cord using an Infinite Horizon impactor (Precision Systems and Instrumentation, LLC, Kentucky, USA) between C6 and C8 aligned medially to the dorsal vein. Dorsal arachnoiditis was subsequently induced by injecting a 5 µL suspension of kaolin in sterile saline (500 mg/mL) into the subarachnoid space at the injury site. The wound was closed with 4-0 Absorbable Coated Vicryl sutures (Ethicon, Johnson & Johnson Medical Pacific Pty Ltd, Sydney, Australia). Buprenorphine (0.05 mg/kg of 324 µg/mL subcutaneous) was administered post-operatively, with subsequent doses given twice daily for up to 5 days. Sutures were removed 7 days post-surgery under anesthesia.

### AQP4 immunofluorescence microscopy

#### Perfusion-fixation

At 12 weeks after surgery, animals were anesthetized using 5% isoflurane in oxygen (1 L/min) and maintained with 2–3% isoflurane in oxygen (0.2 L/min). Animals were intracardially perfused with 1250 IU heparin in 100 mL ice-cold 0.1 M phosphate-buffered saline (PBS), pH 7.4, followed by 200 mL ice-cold solution of 3% paraformaldehyde and 2.5% glutaraldehyde in sodium phosphate buffer (0.1 M), pH 7.2. The spinal cords were extracted, segmented and post-fixed overnight. On the following day, spinal cord segments C2–T3 were cryoprotected with 30% sucrose in 0.1 M PBS for 24–48 h, embedded in Optimal Cutting Temperature compound (OCT, ProSciTech Pty Ltd, QLD, Australia) and frozen at − 80 °C.

#### Immunofluorescence labelling

Using a cryostat (Leica CM 1950 Cryostat, Amtzell, Germany), 20 µm sections of spinal segments C2–T3 were mounted onto Superfrost glass slides (ThermoFisher Scientific, MA, USA) and stored overnight at 4 °C. Subsequently, slides were dried at 37 °C for 1 h and treated with sodium borohydride (1%) for 15 min to break aldehyde covalent bonds. Tissue was permeabilized in 50% ethanol (20 min) and blocked for 1 h (10% normal horse serum, 5% bovine serum albumin, 200 mM glycine). The slides were incubated overnight with a 1:200 dilution of AQP4 antibody (A5971, Merck, Australia) in 4% normal horse serum. After 2 h left at room temperature, tissue was incubated with 1:200 donkey anti-rabbit 488 secondary antibody (ab150073, AbCam, Australia) in 4% normal horse serum for 1 h at room temperature. Tissue was then incubated with anti-smooth muscle actin antibody (SMA: 1:200, MA1-06110, ThermoFisher Scientific, MA, USA) for 30 min at 37 °C. Slides were coverslipped and stored overnight at 4 °C before imaging using a fluorescence microscope (Z2, Zeiss, Oberkochen, Germany).

#### Image and statistical analysis

Fluorescence micrographs were captured at the level of the syrinx in PTS tissue (n = 4) and the respective spinal cord segment in control tissue (n = 4). Qualitative and quantitative assessment of immunofluorescence was performed using ImageJ software^47^. Integrated density of AQP4 expression was calculated over regions of interest (ROIs) at syrinx borders, examined over three spinal cord replicates per animal. Syrinx area was measured in ROIs. A Pearson’s Correlation Test was performed to determine the correlation between AQP4 expression and syrinx area in GraphPad software (Prism 8, San Diego, CA 92108, USA). *P*-values < 0.05 were considered significant and measurements were presented as mean ± SD.

### AQP4 immunoelectron microscopy

#### Perfusion-fixation

Animals were perfusion-fixed under anesthesia as described above, with the fixative substituted with a solution of paraformaldehyde (4%) and glutaraldehyde (0.5%) in sodium phosphate buffer (0.1 M), pH 7.2. The spinal cords were extracted, segmented and post-fixed overnight in the fixative solution. On the following day, 1 mm transverse sections were collected from spinal cord segments C5–T1 and tissue was prepared for transmission electron microscopy (TEM).

#### Sample preparation

Sample preparation followed a previously detailed method^48^, with the exception of heavy metal post-fixation. In short, 1 mm transverse spinal cord sections (C5–T1) were dehydrated with graded ethanol solutions and infiltrated with LR White resin (ProSciTech, Queensland, Australia) in decreasing ethanol dilutions. Further infiltration used pure LR White resin under − 80 kPa of pressure and tissue was embedded and polymerized in gel capsules. Resin blocks underwent ultramicrotome sectioning (Leica EM UC7, Wetzlar, Germany). Tissue was oriented using 1% methylene blue stained semi-thin sections (750 nm), and then triplicate, consecutive ultra-thin sections (70–80 nm) were mounted on formvar-coated 150-mesh nickel grids and dried.

#### Immunoelectron microscopy

Immunogold labelling of AQP4 was performed on-grid and was based on a previously published method^49^. Briefly, matched grids for PTS and control tissue were prepared in triplicate and floated on 50 mM glycine in PBS and blocked with 2% normal goat serum in PBS. Overnight, grids were incubated at room temperature with an AQP4 antibody (A5971, Merck, Australia) at a 1:200 dilution in blocking solution. Secondary blocking occurred prior to room temperature incubation with a 1:20 dilution of anti-rabbit IgG conjugated to 10 nm gold nanoparticles (G-7402, Sigma, Australia) in blocking solution. Sections on-grid were contrast enhanced by incubation with uranyl acetate replacement stain (2 h) and Reynold’s lead citrate (150 s) and allowed to dry overnight. Grids were deidentified for blinded analysis and imaged using a Megaview G2 digital camera (Olympus SIS, Münster, Germany) attached to a Philips CM10 TEM.

#### Image and statistical analysis

AQP4 expression was quantified in astrocytes at the glia limitans, and surrounding arterioles, venules, and capillaries, using ImageJ software^47^. ROIs were drawn around the glial-vessel and glial-pial borders and converted to binary files. The 10 nm particles were isolated by manual thresholding, which allowed total expression of AQP4 to be quantified. The perimeters of vessels and the glia limitans were delineated in 13,500 × micrographs and used to create ROI sets at a defined width. Expression of AQP4 at both vessel and glia limitans were presented as a ratio of perimeter, as perimeter was a consistent measure along the astroglial surface in each 13,500 × micrograph examined. AQP4 expression in groups (arterioles, venules, capillaries, and glia limitans) were compared with unpaired t-tests. Measurements are presented as mean ± SD with P-values < 0.05 considered significant.

### AQP4 activity modulation

#### Aquaporin modulators

Modulation of AQP activity was achieved in vivo using the AqF026 agonist (0.652 mg/kg) derived from a furosemide, and a synthetic small molecule AqB050 antagonist (0.682 mg/kg) based on a bumetanide. The drugs were dissolved in the vehicle (5 mL/kg), 1% dimethyl sulfoxide (DMSO) in sterile saline.

#### Magnetic resonance imaging

MRI was performed in vivo using a Bruker Biospec 9.4-Tesla MRI scanner at the Biological Resources Imaging Laboratory at the University of New South Wales. A semi-circular, polarized, whole-body radiofrequency, 72 mm^3^ quadrature coil was used for radiofrequency transmission and reception. Baseline T2-weighted MRI was performed 6 weeks after syrinx induction, before the commencement of pharmacological modulation of AQP4. A second MRI was performed 5 days later after four once-daily intraperitoneal injections of AQP modulator or vehicle.

Animals were placed under general anesthesia using 5% isoflurane in oxygen (1 L/min) and maintained through a nose cone with 2–3% isoflurane in oxygen (0.2 L/min) during MRI acquisition. The animal was placed prone, and the head stabilized with a tooth bar, with the syrinx centred within the receiving coil. Respiration and heart rate were monitored during imaging. A warming mat set at 36 °C was placed under the animal to maintain body temperature. MRIs were acquired in both sagittal and axial planes using a rapid acquisition with relaxation enhancement (turbo RARE) method. Sequence parameters for sagittal acquisition were as follows: TE (effective) 26 ms, TR 2200 ms, slice thickness 0.5 mm, slice gap 0 mm, field of view (FOV) 50 × 30 mm, acquisition matrix 284 × 384 mm, in-plane resolution 130 × 130 μm, and 3 signal averages. Sequence parameters for axial acquisition were as follows: TE 18 ms, TR 2000 ms, slice thickness 0.5 mm, slice gap 0 mm, FOV 20 × 20 mm, acquisition matrix 150 × 150, in-plane resolution 133 × 133 μm, and 18 signal averages. The duration of each MRI acquisition was approximately 60 min.

#### MRI syrinx volume quantification

Syrinx volumes pre- and post-modulation of AQP4 were quantified using axial MR images processed in ImageJ^47^. ROIs were defined by manually tracing the spinal cord and calculating total area. Common anatomical landmarks (airways and muscles) were used to acquire the mean grey values required for background subtraction and normalization, respectively. The hyperintense syrinx cavity was delineated from grey and white matter by manually selecting a threshold. Pixel value was then used to calculate voxel volume and subsequently syrinx volume in mm^3^ for each MRI slice. Absolute syrinx volume was calculated by multiplying the total syrinx pixel count by voxel volume. Relative syrinx volume was then calculated as a percentage of whole spinal cord volume for the total syrinx length. Using the sagittal MRIs, maximum syrinx length was quantified by measuring the maximum length of the hyperintense region within the cord.

#### Perfusion-fixation and cryoprotection

At 12 weeks after PTS induction surgery, animals were perfusion-fixed under anesthesia as described above, with the fixative substituted with paraformaldehyde (4%) in 0.1 M sodium phosphate buffer, pH 7.4. Spinal cords were extracted and post-fixed overnight. Post-fixed spinal cords were submerged in a cryoprotectant solution of 30% sucrose in 0.1 M sodium phosphate buffer for 24–48 h, and then embedded in cryomolds using OCT compound and frozen at − 80 °C.

#### Histological examination

Using a cryostat (Leica CM 1950 Cryostat, Amtzell, Germany), the embedded spinal cord was mounted and 20 µm transverse sections were serially collected and placed onto Superfrost slides. Serial spinal cord sections of the entire syrinx were stained with hematoxylin and eosin (H&E) and imaged using a digital camera attached to a microscope (Z2, Carl Zeiss, GmbH, Germany). Using ImageJ software^47^, the spinal cord was manually segmented and automatically thresholded to isolate the syrinx cavity of each serial section. Total syrinx volume was calculated across all serial sections (area of syrinx multiplied by section thickness). Total syrinx volume was also normalized against total cord volume. The number of serial sections multiplied by section thickness (20 µm) gave syrinx length.

### Conference presentation

Portions of this work were presented at the Adelaide Centre for Spinal Research Symposium, Adelaide, Australia, 2018, the Syringomyelia-Chiari International Symposium, Birmingham, United Kingdom, 2018, and the CSF Flow at Niagara Falls Conference, Buffalo, New York, USA, 2019.

## Data Availability

The data generated during and/or analyzed during this study are available from the corresponding author on reasonable request.
